# Rapid Divergence of Wing Volatile Profiles Between Subspecies of the Butterfly *Pieris rapae* (Lepidoptera: Pieridae)

**DOI:** 10.1093/jisesa/iey026

**Published:** 2018-03-23

**Authors:** Eden W McQueen, Nathan I Morehouse

**Affiliations:** 1 Department of Biological Sciences, University of Pittsburgh, Langley Hall, Pittsburgh, PA; 2 Department of Biological Sciences, University of Cincinnati, Cincinnati, OH

**Keywords:** pheromones, speciation, allopatry, Lepidoptera

## Abstract

Complex signaling traits such as pheromone profiles can play an important role in the early stages of reproductive isolation between populations. These signals can diverge along multiple trait axes, and signal receivers are often sensitive to subtle differences in signal properties. In the Lepidoptera, prior research has highlighted that natural selection can drive rapid chemical signal divergence, for instance via mate recognition to maintain species boundaries. Much less is known about the occurrence of such changes for predominantly sexually selected chemical signals, such as those released by many male lepidopterans. We evaluated the divergence in male and female wing volatile profiles between two recently isolated subspecies of the pierid butterfly *Pieris rapae* Linnaeus (Lepidoptera: Pieridae): *P. rapae rapae* and *P. rapae crucivora*. In laboratory settings, these subspecies exhibit strong premating isolation, with females rejecting males of the opposite subspecies despite the fact that males direct equivalent courtship effort toward females of either subspecies. Using gas chromatography–mass spectrometry, we analyzed the volatile chemical profiles of individual males and females of each subspecies. We find that males of each subspecies differ in their wing volatile profiles, including quantitative differences in a male sex pheromone, ferrulactone. In contrast, female wing volatiles profiles have diverged significantly less. These sex-specific patterns suggest that male chemical profiles may play a role in the observed premating isolation between these two subspecies, providing support for future investigations of sexually selected chemical traits in population divergence.

Examining how and why reproductive isolating barriers arise is central to our understanding of speciation processes (Coyne and Orr 2004). Sexual selection can lead to strong yet unpredictable directional selection on reproductive traits, which can result in the evolution of prezygotic isolating barriers between populations much more quickly than would be expected by neutral processes such as drift (Andersson 1994, Panhuis et al. 2001). A better grasp of the timescales upon which sexual traits evolve in allopatry should provide fundamental insights into how isolated populations might begin to speciate. However, these changes in the fleeting early stages of divergence can be difficult to observe.

Pheromones are promising candidates for studying early divergence in sexual signaling. Chemical signals can be quite evolutionarily labile, due to both the multi-component nature of pheromone profiles and the rapid evolution of the biosynthetic pathways from which they are generated (Carde and Haynes 2004, Roelofs et al. 2002, Symonds and Elgar 2008, Fang et al. 2009, Shirangi et al. 2009, Palmer et al. 2010, Wyatt 2014). In addition, olfactory and gustatory systems are enormously complex, and these too are known to diversify quickly (e.g., Palmer et al. 2005, Sánchez-Gracia 2009, Vieira and Rozas 2011, Bousquet et al. 2012). Even subtle differences in pheromone blend composition and compound ratios are known to be detectible by insects in many systems, often with behavioral consequences (Raguso 2008, Wyatt 2014).

In the Lepidoptera, the evolution of chemical signals and the role of pheromones in reproductive isolation have received much attention (Symonds and Elgar 2008, Smadja 2009, Allison and Cardé 2016). Most of these studies focus on the female-released pheromones of moths, which are primarily used by males for mate identification and mate location. From these studies, we know that chemical profiles can diverge very rapidly between allopatric populations, even on short timescales (e.g., Löfstedt 1986, Allison and Cardé 2007). However, these signals are strongly influenced by natural selection due to their role in species identification (Allison and Cardé 2016). Much less attention has been paid to the evolutionary divergence of male courtship pheromones in the Lepidoptera. Current evidence suggests that these male pheromones are often subject to sexual selection via female choice, rather than, or in addition to, species identification (Rutowski 1984, Löfstedt et al. 1991, Iyengar et al. 2001, Costanzo and Monteiro 2007, Nieberding et al. 2008, Lassance and Löfstedt 2009). Thus, at least for the Lepidoptera, we know relatively little about how sexual selection might be involved in the evolution of pheromones during the initial stages of population divergence.

Here, we used two subspecies of the Cabbage White butterfly *Pieris rapae* (Lepidoptera: Pieridae) to investigate whether the volatile chemicals produced on the wings of males and females have diverged during a short period of allopatry. The European subspecies *P. r. rapae* and the Japanese subspecies *P. r. crucivora* are estimated to have become isolated after a rapid eastward expansion from Europe across the Asian continent and subsequent colonization of Japan approximately 5,000 yr ago (Fukano et al. 2012). The subspecies have yet to evolve significant post-zygotic genetic barriers (crosses produce viable, fertile offspring: Obara et al. 2010). However, attempts to cross these subspecies in the laboratory result in low mating rates between subspecies as compared to within subspecies (N. Saleh, personal communication, Obara and Majerus 2009). Males of both subspecies vigorously court females from either group, but females typically reject males of the opposite subspecies. These preliminary observations suggested that males and/or females may have diverged between the subspecies in either their sexual traits or associated preferences. We focused our attention on chemical signaling for the reasons described in what follows.

Courtship in *P. rapae* occurs in two stages. During early courtship, males pursue flying females and perform aerial courtship behaviors that showcase colors on the dorsal surfaces of their wings (Suzuki 1977, Morehouse and Rutowski 2010). If the female chooses to land, the second stage of courtship consists of the male approaching her on the substrate, at which point she chooses to either accept or reject him (Suzuki 1977). Mate recognition in butterflies during the first stage is thought to be primarily based on visual cues (Rutowski 1991, Vane-Wright and Boppré 1993, Wiklund 2003, Morehouse and Rutowski 2010), whereas mate assessment during the second stage is thought to also be mediated predominantly by chemical signals (Vane-Wright and Boppré 1993). The small enclosures we use for laboratory matings largely restrict male–female interactions to the later stages of courtship, which, therefore, favors chemical signaling. In addition, although the subspecies have diverged in wing coloration (Morehouse and Rutowski 2010, Tigreros et al. 2014), visual modeling based on *P. rapae* color sensitivities reveals that females differ dramatically in color appearance between the subspecies, but males do not (N. Morehouse, unpublished data). Consistent with this female-limited divergence in coloration, Obara and Majerus (2000) described subspecific differences in male recognition of female mates, but no such subspecific differences have been described for female responses to male coloration. Thus, we think it is unlikely that subtle differences in male wing coloration between the subspecies are responsible for the dramatic premating isolation observed in laboratory crosses. Current evidence suggests that male pheromones are often the focus of sexual selection via female choice (Wiklund 2003, Andersson et al. 2007), whereas males may rely more heavily on visual cues (Obara and Majerus 2000). Given that females are rejecting males in this case, we chose to investigate the volatile chemicals of these two subspecies as a potential mode of mate discrimination.

Previous work by Yildizhan et al. (2009) characterized the full volatile chemical profile of male and female *P. rapae rapae* using mass spectrometry of wing extracts pooled from many individuals. They then demonstrated that both sexes can detect at least a subset of these compounds, and further, that several of the compounds identified from male wings play a role in male courtship success and, therefore, function as male sex pheromones (Yildizhan et al. 2009). Building on this prior work, we characterized and compared the wing chemical profiles of individual males and females of both *P. r. rapae* and *P. r. crucivora*, to elucidate whether the volatile chemical profiles, especially with regard to known pheromones, have diverged in allopatry between these subspecies.

## Materials and Methods

### Experimental Animals

Male and female *P. r. rapae* and *P. r. crucivora* were laboratory reared progeny of wild-caught females collected during the same field season. Wild-caught *P. r. rapae* females were collected in Rochester, Pennsylvania (40°44′45″N, 80°9′45″W) in May and June 2014. *P. r. crucivora* females were collected from field sites near Sokendai, Miura District, Kanagawa, Japan (35°15′47″N, 139°36′32″E) in April 2014, and their offspring were shipped as eggs to the University of Pittsburgh (authorized under USDA permit P526P-15-00485). All life stages were housed in climate-controlled chambers maintained at 24 ± 0.1°C, 60% relative humidity, and a 16:8 (L:D) h photoperiod cycle. Caterpillars were reared in small groups on kale [*Brassica oleracea* var*. acephala* L. (Brassicales: Brassicaceae)] grown in a greenhouse. Pupae were removed from caterpillar-rearing containers and kept in individual cups until eclosion. Upon eclosion, adult males and females were housed individually in small containers and fed daily on 25% honey water for 5 d. On Day 5 post-eclosion, butterflies were euthanized at −80°C, between 12:00 and 14:00 EST. The volatile chemical compounds of the wing surface, where their pheromones are produced (Yildizhan et al. 2009), were immediately extracted. Both the forewings and hindwings of each individual were used.

### Wing Chemical Extraction

We evaluated the wing volatile chemical profiles of 11 males and 11 females of each subspecies (*n* = 44). For each individual, forewing length was measured to the nearest 0.01 mm using digital calipers (Absolute 500 Digimatic Caliper, Mitutoyo, Aurora, IL). Wings were excised from the body using surgical scissors, which were cleaned with ethanol and dried between each use. Wing material was weighed to the nearest 0.1 mg (XS205 Analytical Balance, Mettler-Toledo, Columbus, OH), and then placed in a 4 ml borosilicate glass sample vial with a PTFE-lined cap (Wheaton, W224582). Five hundred microliters of CH_2_Cl_2_ (Merck, Suprasolv, Kenilworth, NJ) were added to the vial and the material was extracted at room temperature. Vials were periodically shaken gently during extraction, but wing material was kept as intact as possible. After 20–30 min, the extract was removed to a second 4 ml vial and stored at −80°C. Once all samples had been extracted, samples were removed from the freezer and prepared for analysis. Two hundred and fifty microliters of extract were transferred to a glass analytical vial (National Scientific, MSCERT4000-31LVW, Waltham, MA) through a glass Pasteur pipet plugged with quartz wool to filter out solid tissue. Ten microliters of 0.01% toluene (Sigma Aldrich, Chromasolv, St. Louis, MO) were then added as an internal standard (IS). The toluene standard was used as a reference of known initial concentration so that peak profiles could be normalized and compared across samples. The solvent was dried down using a light flow of filtered air to a volume of 20–50 μl. Some percentage of volatile components, including a fraction of the IS, may have been lost during concentration. This could result in over or under estimation of initial IS to focal compound ratio, depending on the end sample volume. However, as the same procedure was used for each vial, any such loss would be random with respect to sample group. Vials were capped and immediately analyzed by gas chromatography–mass spectrometry (GC–MS).

### Analytical Methods

All samples were analyzed by GC–MS (Shimadzu, GCMS-QP2010S, Kyoto, Japan) using a SHRXI-5MS fused silica capillary column (Shimadzu, 30 m × 0.25 mm × 0.25 μm, Kyoto, Japan), with helium as the carrier gas. The program was set to hold the temperature at 70°C for 1 min, then increase the temperature by 4.00°C/min to 270°C, which was then held for 15 min. Blank injections were run between each sample. Peak areas for most components were obtained through automated peak integration using the GCsolution workstation (Shimadzu Corporation). Visible peaks which fell below the detection threshold of the automated program were manually integrated using the same software. Peaks were identified using their mass spectra, as well as the elution order and identities of compounds known to be produced by *P. r. rapae* from a previous study (Yildizhan et al. 2009). The identity and retention time of heptacosane under this method were validated using an authentic standard (Sigma Aldrich).

### Statistical Analyses

All statistical analyses were completed using R version 3.0.2 (R Core Team 2013). For all analyses, peak areas were normalized by dividing the peak area of the compound by the peak area of the IS, generating a unitless ratio that was used to describe each compound in terms of relative amount. Group means and standard errors were calculated for each compound using the normalized values. When appropriate, effect sizes were estimated as either Hedge’s *g*_*s*_ or omega-squared (ω^2^) statistics following formulas in Olejnik and Algina (2003).

We compared the chemical profiles of male and female *P. r. rapae* and *P. r. crucivora* by performing one-way analysis of variance (ANOVA) for each compound. Because differences in body size alone could be responsible for observed differences in chemical production between the subspecies, we evaluated whether male *P. r. crucivora* differed from male *P. r. rapae* in wing mass or forewing length. However, no significant differences in male body size were observed between these two groups (Student’s *t* -tests: wing mass, *t* = 0.9028, df = 20, *P* = 0.377, Hedge’s *g*_*s*_: 0.3704; forewing length, *t* = 1.779, df = 18.70, *p* = 0.09158, Hedge’s *g*_*s*_: 0.7388). However, female body size did differ (Student’s *t*-tests: wing mass, *t* = 0.9028, df = 20, *P* < 0.001, Hedge’s *g*_*s*_: 0.3704; forewing length, *t* = 1.779, df = 18.89, *P* < 0.001, Hedge’s *g*_*s*_: 0.7388). Therefore, wing mass was included as a covariate for the female analyses.

Multivariate analyses were used to investigate differences in chemical profiles between all four groups (male *P. r. rapae*, female *P. r. rapae*, male *P. r. crucivora*, and female *P. r. crucivora*). The analyses (described in detail subsequently) were performed on four variations of the dataset.

First, the multivariate analysis was performed using the IS normalized values for all identified compounds (hereafter ‘Analysis 1’). A second analysis (hereafter ‘Analysis 2’) was then performed using only compounds shown to be detectable to *P. r. rapae* from previous studies (Yildizhan et al. 2009, Li and Mathews 2016). The detectability of compounds is noted in Table 1. Examining this subset of the data was done to provide greater confidence that differences discovered between groups were attributable to compounds that are known to be detectable by these animals, and, therefore, more plausibly involved in signaling. We note that we chose to first perform the full analysis (using all compounds) due to the possibility that some compounds that were not definitively shown to be detectable by GC-EAD in a prior study (Yildizhan et al. 2009) might still be detectable to these animals in the wild. Also, some of the compounds not known to be detectable by *P. r. rapae* could be detectable by *P. r. crucivora*. Lastly, it is possible that the nature of lab-based assays (e.g., GC-EAD) might affect the volatility or perceptibility of components of the chemical blend, and, therefore, indirectly affect detectability.

**Table 1.** Means (%) ± standard deviations of 13 major components in male and female wings, as unitless normalized ratios to the internal standard

No.	Compound	GC-EAD signal?	Group				Difference between subspecies MALES	Difference between subspecies FEMALES^*a*^
			*P. r. rapae* Male	*P. r. crucivora* Male	*P. r. rapae* Female	*P. r. crucivora* Female		
1	Indole	N	0.119 ± 0.084	0.034 ± 0.025	not present	not present	**	NA
2	Ferrulactone	Y	2.206 ± 0.735	0.714 ± 0.299	not present	not present	***	NA
3	Hexahydrofarnecyl acetone	Y	4.434 ± 3.479	0.910 ± 0.719	not present	not present	**	NA
4	Phytol derivative	N	0.086 ± 0.067	0.004 ± 0.006	not present	not present	***	NA
5	Phytol	Y	1.047 ± 0.776	0.264 ± 0.288	not present	not present	**	NA
6	Tricosane	Y	0.013 ± 0.021	0.039 ± 0.038	0.714 ± 0.697	2.577 ± 2.852	ns	**
7	Pentacosane	Y	0.110 ± 0.075	0.260 ± 0.172	0.386 ± 0.207	2.099 ± 2.346	ns	**
8	Hexacosane	N	0.009 ± 0.016	0.022 ± 0.037	0.039 ± 0.029	0.141 ± 0.115	ns	*
9	Heptacosane	Y	1.612 ± 0.991	2.303 ± 1.555	2.779 ± 1.046	4.542 ± 1.588	ns	*
10	Octacosane	N	0.102 ± 0.074	0.128 ± 0.091	0.134 ± 0.048	0.179 ± 0.048	ns	ns
11	Nonacosane	Y	3.172 ± 1.569	4.390 ± 2.606	4.126 ± 1.423	5.202 ± 1.387	ns	*
12	Hentriacontane	N	0.139 ± 0.079	0.215 ± 0.109	0.255 ± 0.100	0.437 ± 0.208	ns	**
13	Cholesterol	N	0.483 ± 0.284	1.426 ± 0.763	0.167 ± 0.147	0.291 ± 0.232	***	ns

Compounds demonstrated by Yildizhan et al. (2009) to be detectable by the butterflies via GC-EAD are indicated. Significant differences between subspecies are also noted. Some compounds that occurred in trace amounts were identified based on relative retention times of known components of the chemical profile from this species, as the molecular ion was not able to be detected. See text.

^*a*^Female analysis of variance includes forewing length as a covariate to correct for differences in female body size across subspecies.

Significance levels: ***P ≤ 0.001, **P ≤ 0.01, *P ≤ 0.05.

The presence and quantity of a particular chemical compound detected by a receiver would likely be based on overall wing content. However, we were also interested in investigating whether there are any changes to the concentration of volatile wing chemicals per unit wing tissue above and beyond a divergence in wing size, as the size of the wings could be subject to selection apart from selection on chemical production. We reasoned that correcting for mass would highlight changes due to, for example, increased or decreased chemical production per androconial (pheromone-producing) cell, or increased or decreased size of androconial cell patches on the wings, which have not been characterized for *P. rapae,* and could vary subspecifically. Therefore, two additional multivariate analyses (‘Analysis 3’ and ‘Analysis 4’) were conducted using mass-adjusted values. The third analysis was a mass-adjusted repeat of the first analysis (using all identified compounds) and the fourth was a mass-adjusted repeat of the second analysis (using only compounds of known detectability). To adjust for mass, the normalized value of each compound was divided by that individual’s wing mass prior to analysis.

All four datasets were analyzed as follows. First, a principal component analysis (PCA) was performed using the normalized values for all of the identified compounds. To determine whether the four groups (*P. r. rapae* females, *P. r. rapae* males, *P. r. crucivora* females, *P. r. crucivora* males) differ in principal component space, a MANOVA was performed using the first three principal components as dependent variables in a model that included all four groups. This was followed by a series of Bonferroni-corrected pairwise comparisons.

To determine how well the four groups could be distinguished in multivariate space using a supervised technique, a linear discriminant analysis (LDA) was performed and then assessed for robustness using leave-one-out cross-validation. Mahalanobis distances (MD) were calculated to quantify the degree of separation observed in the discriminant analysis. Bonferroni-corrected pairwise MANOVAs were performed to look for differences between groups, using the first three linear discriminants (LD) as dependent variables.

## Results

### Chemical Analyses and Statistical Comparison of Male Chemical Profiles

**Fig. 1.**
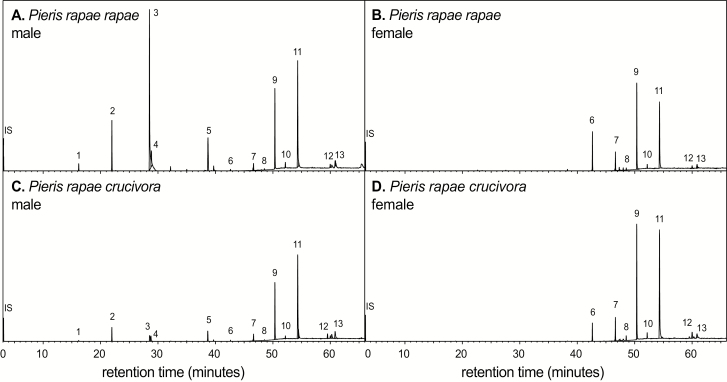
Typical chromatograms from wing extracts of single individuals. (A) *Pieris rapae rapae* male. (B) *P. r. rapae* female, C. *P. r. crucivora* male, D. *P. r. crucivora* female. Compound identities are as follows: (1) Indole, (2) Ferrulactone, (3) hexahydrofarnecyl acetone, (4) Phytol derivative, (5) Phytol, (6) Tricosane, (7) Pentacosane, (8) Hexacosane, (9) Heptacosane, (10) Octacosane, (11) Nonacosane, (12) Hentriacontane, (13) Cholesterol.

**Fig. 2.**
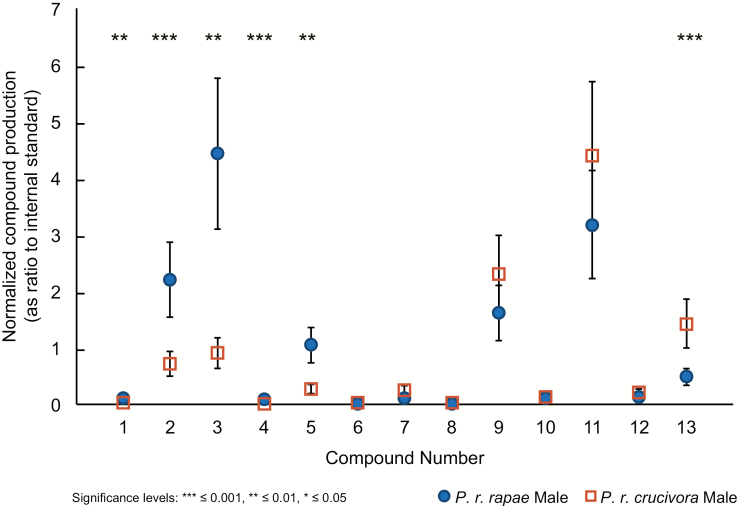
Plot of subspecies differences in compound level for males. Significance of difference between compound levels is as noted by asterisks (*0.05 > *P* > 0.01, **0.01 > *P* > 0.001, ***P < 0.001). Compound identities are as follows: (1) Indole, (2) Ferrulactone, (3) hexahydrofarnecyl acetone, (4) Phytol derivative, (5) Phytol, (6) Tricosane, (7) Pentacosane, (8) Hexacosane, (9) Heptacosane, (10) Octacosane, (11) Nonacosane, (12) Hentriacontane, (13) Cholesterol. Values are listed in Table 1.

Thirteen compounds in the chemical blends were detectable by GC–MS in all individuals in quantities sufficient for analysis (Table 1). Figure 1 shows a typical chromatogram of wing extracts from an individual belonging to each of the four groups. Table 1 includes a column indicating which of the identified compounds were shown to be detectable by *P. r. rapae* in a previous study using GC-EAD (Yildizhan et al. 2009). None of these 13 compounds were found to occur only in females, nor were any of these compounds found exclusively in one subspecies. Higher straight chain alkanes (**6–12**) were the predominant compounds produced by all groups, as well as cholesterol (**13**, characteristic ion at *m*/*z* 368). Several compounds (**1–5**) were only found in males. Figure 2 plots the chemical profiles of males for each subspecies, showing the quantities produced for all 13 compounds. Indole (**1**, *m*/*z* 117) is used by *P. r. rapae* as a component of an anti-aphrodisiac blend (Andersson et al. 2003). **1** was produced in small amounts by the males of both subspecies, but the level produced by *P. r. crucivora* males was statistically lower (ANOVA: *F*_1,20_ = 10.34, *P* = 0.004, ω^2^ = 0.8236). Levels of ferrulactone (**2**, *m*/*z* 194) were significantly higher in *P. r. rapae* males than *P. r. crucivora* males (ANOVA: *F*_1,20_ = 38.94, *P* < 0.001, ω^2^ = 0.9499). The mass spectrum for **3** was not definitive (molecular ion peak was not detected for any sample). However, only one compound previously identified for this species, hexahydrofarnecyl acetone, is produced in the observed abundance in this region of the spectrum (based on the position of this peak relative to peaks of known identity); therefore, this peak was tentatively identified as hexahydrofarnecyl acetone (**3**). One of the male compounds appeared in most individuals but could not be conclusively identified. Based on the spectrum, this compound appears to be terpenoid derivative of phytol (**5**, characteristic ion at *m*/*z* 278). The component was thus labeled ‘phytol derivative’ (**4**). All three compounds (**3**, **4**, and **5**) are produced in statistically greater quantities by *P. r. rapae* males than *P. r. crucivora* males (ANOVA: **3**: *F*_1,20_ = 10.82, *P* = 0.004, ω^2^ = 0.8308; **4**: *F*_1,20_ = 16.52, *P* < 0.001, ω^2^ = 0.8858; **5**: *F*_1,20_ = 9.843, *P* = 0.005, ω^2^ = 0.8155). **13** was produced in greater quantities by *P. r. crucivora* males (ANOVA: *F*_1,20_ = 14.77, *P* = 0.001, ω^2^ = 0.8732). Levels of the remaining components, (**6**, tricosane, *m*/*z* 324; **7**, pentacosane; **8**, hexacosane; **9**, heptacosane, *m*/*z* 380; **10**, octacosane; **11**, nonacosane, *m*/*z* 408; and **12**, hentriacontane) all *n*-alkanes, did not significantly differ between males of the two subspecies (we note that the molecular ions of trace components could not always be detected, but rather the identities of these components were determined using the known presence and order of these compounds in the *P. rapae* chemical profile, and characteristic *n*-alkane ions in their mass spectra).

### Multivariate Analyses of Population and Sex Differences in Chemical Profiles

The results of the PCA revealed similar patterns for all four dataset variations. In all cases, a MANOVA using the first three principle components detected significant differences when group identity was used as the predictor (Table 2). All subsequent pairwise MANOVAs yielded significant differences between all specific group pairs for all four variants of the analysis except for the two female groups (Table 2). A significant difference between female *P. r. rapae* and *P. r. crucivora* could only be detected in the analysis using all identified compounds and no body size correction.

**Table 2.** Results of MANOVA on the first three components from the principle component analysis of chemical profiles

	*df*	Wilkes *λ*	*F*	*P*-value^*a*^
1. All identified compounds, no body size correction				
All four groups	3	0.120	16.5	<2.20 E -16
RM × CM	1	0.302	13.9	3.77E-04
RM × RF	1	0.159	35.3	2.08E-07
CM × RF	1	0.197	27.2	1.74E-06
CM × CF	1	0.245	22.6	3.89E-06
RM × CF	1	0.152	40.8	2.20E-08
RF × CF	1	0.608	5.16	0.0408
2. Known detectable compounds, no body size correction				
All four groups	3	0.124	16.2	<2.20 E -16
RM × CM	1	0.323	12.6	0.000674
RM × RF	1	0.162	34.5	2.55E-07
CM × RF	1	0.284	18.5	1.91E-05
CM × CF	1	0.367	11.5	0.000809
RM × CF	1	0.127	50.5	2.97E-09
RF × CF	1	0.686	3.66	0.159
3. All identified compounds, with body size adjustment				
All four groups	3	0.0879	20.4	<2.20 E -16
RM × CM	1	0.246	18.4	6.09E-05
RM × RF	1	0.0964	62.5	1.47E-09
CM × RF	1	0.200	26.7	2.01E-06
CM × CF	1	0.207	28.1	6.30E-07
RM × CF	1	0.118	55.0	1.32E-09
RF × CF	1	0.651	4.28	0.0887
4. Known detectable compounds, with body size adjustment				
All four groups	3	0.0904	20.1	<2.20 E -16
RM × CM	1	0.262	16.9	1.07E-04
RM × RF	1	0.101	59.0	2.45E-09
CM × RF	1	0.308	15.0	1.44E-04
CM × CF	1	0.278	19.1	1.52E-05
RM × CF	1	0.109	60.0	5.63E-10
RF × CF	1	0.785	2.19	0.692

RF = *P. r. rapae* females, RM = *P. r. rapae* males, CF = *P. r. crucivora* females, CM = *P. r. crucivora* males.

^*a*^Bonferroni adjusted.

The loadings of each compound into the first three principal components for Analysis 1 are shown in Table 3 (see Supp Table 1 [Online only] for the loadings for a table of all four analyses). PC1 was negatively correlated with compounds **1–5** in all analyses that included some or all of these compounds. PC1 was slightly negatively correlated with compound **13** for the two analyses that included **13**.

**Table 3.** Principle component loadings of 13 major components extracted from wings of males and females, not adjusted for body size

No.	Compound	PC1 (49.77%)	PC2 (24.60%)	PC3 (11.49%)
1	Indole	-0.28	-0.40	0.02
2	Ferrulactone	-0.33	-0.24	-0.10
3	Hexahydrofarnecyl acetone	-0.28	-0.31	-0.07
4	Phytol derivative	-0.26	-0.32	-0.12
5	Phytol	-0.28	-0.39	-0.02
6	Tricosane	0.30	-0.18	-0.46
7	Pentacosane	0.29	-0.21	-0.46
8	Hexacosane	0.33	-0.25	-0.24
9	Heptacosane	0.33	-0.27	0.08
10	Octacosane	0.25	-0.34	0.28
11	Nonacosane	0.22	-0.30	0.45
12	Hentriacontane	0.29	-0.10	0.27
13	Cholesterol	-0.02	-0.10	0.36

Results shown are from Analysis 1 (see Statistical Analyses).

**Fig. 3.**
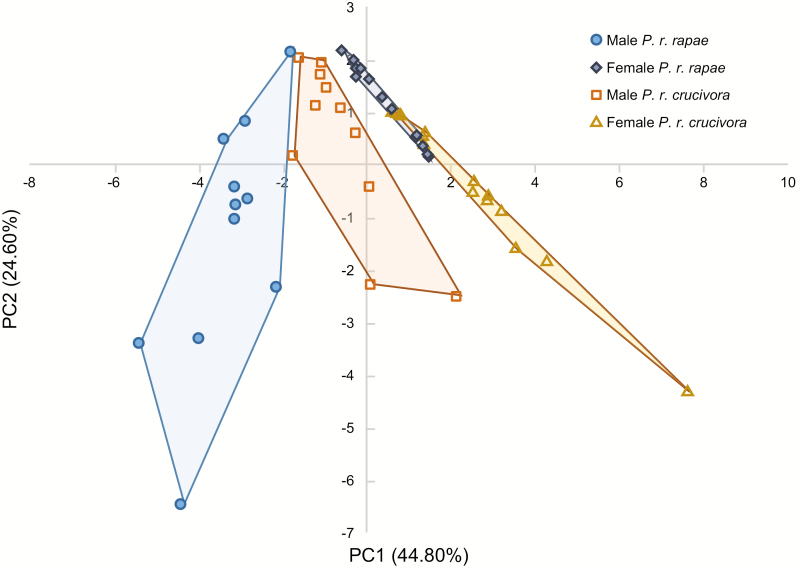
Plot of first two principal components for males and females of both subspecies. PCA revealed significant differences in the pheromone profiles for the four groups. All six pairwise MANOVAs demonstrated statistically significant differences (*P* < 0.05). Convex hulls are included to illustrate the spatial separation of the groups. Results shown are from Analysis 1 (see Statistical Analyses).

A plot of the first two principal components for Analysis 1 is shown in Fig. 3. The principal component scores for all analyses are shown in Supp Table 2 (Online only). Compounds **1–5** are all associated only with males, and in all cases the score for PC1 changes sign by sex, indicating that PC1 primarily describes the difference between males and females. For all variations of the analysis, the mean scores of each group for PC2 and PC3, however, differ in sign between subspecies groups, not sex, indicating that these two principal components describe differences between the subspecies.

We were also able to distinguish groups using LDA. Table 4 indicates the percentage of individuals correctly categorized by group based on individual chemical profile for Analysis 1 (see Supp Table 3 [Online only] for a table of all four analyses). Leave-one-out cross-validation is also shown. As an example, for Analysis 1, individual *P. r. rapae* females were correctly classified based on LDA predictions in 92% of cases. 91% of *P. r. rapae* male individuals and 73% of *P. r. crucivora* females were classified correctly, and 100% of *P. r. crucivora* males were correctly classified. In all analyses these percentages were somewhat lower for the leave-one-out cross-validation, mainly for *P. r. crucivora* females and *P. r. rapae* males, which were often misclassified as *P. r. rapae* females and *P. r. crucivora* males, respectively.

**Table 4.** Group prediction using LD for all compounds

Subspecies and sex	% Correct Prediction	Cross-validation
*P. r. crucivora*, female	73%	60%
*P. r. rapae*, female	92%	92%
*P. r. crucivora*, male	100%	91%
*P. r. rapae*, male	91%	73%

Results shown are from Analysis 1 (see Statistical Analyses).

**Fig. 4.**
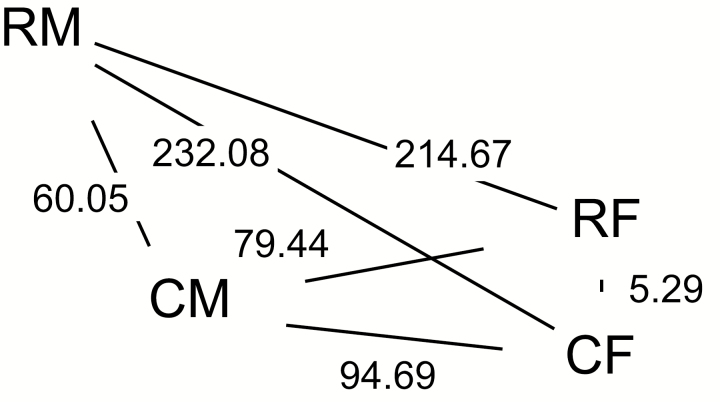
MD calculated using the first three discriminants from the LDA of volatile compositions between groups. MDs were significantly different between all pairs after Bonferroni correction (*P* < 0.05). R indicates the European subspecies, *Pieris rapae rapae*. C indicates the Japanese subspecies *P. r. crucivora*. M is used to designate male, and F is used to designate female. Line segments connecting vertices (but not distances between vertices) are scaled to reflect MDs. Results shown are from Analysis 1 (see Statistical Analyses).

To evaluate differences between groups in multivariate linear discriminant space, we considered differences in the first three LD using MANOVA, followed by a calculation of MD using these three LD. MANOVAs of the first three LD were significantly different between all pairs, even after Bonferroni correction. The results of Analysis 1 are shown in Table 5 (see Supp Table 4 [Online only] for the results of all four analyses). For Analysis 1, both male groups were quite distant (large MD) from both female groups. However, *P. r. rapae* males had a much greater MD separating them from both groups of females (*P. r. rapae* males to *P. r. rapae* females = 214.67, *P. r. rapae* males to *P. r. crucivora* females = 232.08) than did *P. r. crucivora* males (*P. r. crucivora* males to *P. r. rapae* females = 79.44, *P. r. crucivora* males to *P. r. crucivora* females = 94.69). The shortest distance was observed between the two female groups (*P. r. rapae* females to *P. r. crucivora* females = 5.29), whereas the two male groups were intermediately distant (*P. r. rapae* males to *P. r. crucivora* males = 60.05). Figure 4 depicts the MD for all pairings from Analysis 1. For the analyses excluding compounds of unknown detectability, *P. r. crucivora* males were less distant from the two female groups than when all compounds were included (Supp Table 4 [Online only]).

**Table 5.** Mahalanobis distances and MANOVAs of first three linear

Comparison	MD	df	Wilks λ	approx. *F*	*P*-value^*a*^
RM × CM	60.1	1	0.193	25.0	7.25E-06
RM × RF	215	1	0.0888	68.4	6.52E-10
CM × RF	79.4	1	0.117	50.1	1.05E-08
CM × CF	94.7	1	0.0995	66.4	2.09E-10
RM × CF	232	1	0.0788	85.8	1.62E-11
RF × CF	5.29	1	0.548	6.60	0.0124

All identified compounds, not adjusted for body size. Results shown are from Analysis 1 (see Statistical Analyses).

RF = *P. r. rapae* females, RM = *P. r. rapae* males, CF = *P. r. crucivora* females, CM = *P. r. crucivora* males.

^*a*^Bonferroni adjusted.

## Discussion

Our investigation demonstrates that these two closely related subspecies of *P. rapae* have diverged in their volatile chemical profiles over a short evolutionary period (~5,000 yr, or roughly 10–20,000 generations). Both males and females have diverged in their wing volatile chemical composition, but both our principle component analyses and linear discriminant analyses revealed that divergence in volatile profiles was more dramatic for males. Importantly, three of the compounds that differ in amount between subspecies males have been definitively shown to be detectable by these butterflies, and to affect the success of male courtship in *P. r. rapae* (Yildizhan et al. 2009, Li and Mathews 2016). For females, it appears that the observed chemical profile differences are predominantly due to an increase in female *P. r. crucivora* wing size.

It remains to be determined whether females are able to discriminate between males based on the observed shifts in volatile chemical levels we detected here. In *P. r. rapae*, it is known that supplementing the wings of males with a titre of ferrulactone, hexahydrofarnecyl acetone, and phytol (compounds **2**, **3**, and **5**) does generate a behavioral response (Yildizhan et al. 2009). In fact, in both previous studies that investigated pheromones in *P. r. rapae*, addition of pheromone titres resulted in increased mating success, suggesting a ‘more is better’ mechanism of female preference in this species (Yildizhan et al. 2009, Li and Mathews 2016). Given that there is no change in the observed rank order of chemical production between subspecies with the exception of cholesterol (which has previously been shown not to be detectable by *P. rapae*), we might anticipate that *P. r. rapae* males, which have higher quantities of these volatile compounds, would be successful when courting females of either subspecies. Contrary to this prediction, *P. r. crucivora* females are even more discriminatory against heterosubspecific males than females of *P. r. rapae* (Obara and Majerus 2009). However, as the levels of some compounds differ between subspecies and others do not, the *ratios* of detectable compounds do vary between the two groups, an important factor in mate discrimination for many lepidopteran species (e.g., famously, *Ostrinia nubilalis* (Hübner) (Lepidoptera: Crambidae): Klun et al. 1973, Kochansky et al. 1975). For example, for two of the compounds known to generate behavioral response in *P. r. rapae* (Yildizhan et al. 2009), compounds **2** and **3**, have a ratio of 1:2 in *P. r. rapae* males, but a ratio of 1:1.2 in *P. r. crucivora* males.In addition, we do not know whether or how the olfactory sensitivities of *P. r. crucivora* females differ from those of *P. r. rapae* females, which could also contribute to differences in behavior.

The cause of the observed divergence in volatile chemical profiles is not known. However, it is important to note that, given their allopatric distribution, this divergence likely occurred in the absence of secondary contact. This indicates that directional selection resulting from reinforcement between the subspecies cannot explain the observed character divergence. The fact that such strong behavioral isolation has arisen in this system without reinforcement argues for a focus on the roles of sexual selection and drift in this system, processes which have been the subjects of considerable interest with regard to incipient speciation (Lande 1981, Panhuis et al. 2001, Cardé and Haynes 2004, Coyne and Orr 2004, Ritchie 2007, Uyeda et al. 2009). The discovery that the most divergent levels of chemical production are between males of the subspecies, including compounds known to be used for female mate choice in one of the two subspecies, also strongly suggests that sexual selection has contributed to divergence in the male chemical profile. However, behavior assays are needed to determine the functional significance of these differences. In addition, the observations of reproductive isolation between these subspecies that motivated this study were conducted in laboratory mating enclosures. Whether the reluctance of females to mate with males of the opposite subspecies would be realized in the field is uncertain. Interference or masking of chemical signals is often observed in field studies of Lepidoptera (e.g., Lelito et al. 2008, Andersson et al. 2011), and thus even if pheromones are indeed responsible for the premating isolation we observe in this system, it is possible that the laboratory mating conditions produce an unusual level of sensitivity to pheromone differences. Future work identifying the genetic basis of these pheromones could also aid in determining if the observed divergence is the result of directional selection or drift.

Natural selection could be implicated in divergence of long-chain hydrocarbons, which are often associated with ecological functions such as desiccation resistance (Lockey 1988). However, if that were the case we might have expected that the males and females would cluster by subspecies in these compounds, a trend which does not emerge from our data. Natural selection could also be a driver of divergence in the presence of closely related heterospecifics (Higgie et al. 2000), or in the presence of natural predators that eavesdrop on the pheromones of prey or hosts (Coyne and Orr 2004, Symonds and Elgar 2008, Huigens et al. 2010). These possibilities are worth pursuing in future studies of volatile chemical signaling in this species.

There are still many open questions about the involvement of sexually selected traits in speciation processes (Ritchie 2007). Because pheromone profiles are cryptic to human observers, identifying instances of pheromone shifts prior to speciation can present a challenge. A number of case studies in *Drosophila* have shown that cuticular hydrocarbons (CHCs), which are often involved in male and female choice, can diverge between isolated populations of the same species. For example, Rundle et al. (2005) used experimental evolution in *D. serrata* Malloch (Diptera: Drosophilidae) to promote changes to CHCs under different resource conditions. In that species, female mating preferences shifted concurrently, supporting a model of speciation whereby sexual isolation arises through interactions between ecological pressures and sexual selection. Several studies examining populations of *D. montana* Patterson and Wheeler found intraspecific divergence in CHCs with significant effects on mating behaviors (Suvanto et al. 2000, Veltsos et al. 2012, Jennings et al. 2014). To better understand the drivers and effects of intraspecific divergence of sexually selected chemical traits, more examples like these are needed. Species such as *P. rapae*, which appear to be undergoing incipient allopatric speciation, are promising systems for deepening our knowledge on this front.

### Conclusion

Our results demonstrate that the chemical profiles of male *P. rapae* have diverged quantitatively between *P. r. rapae* and *P. r. crucivora* populations during a relatively brief period of allopatry. Other known sexually selected traits in this system vary less dramatically between subspecies, supporting the idea that multi-component traits such as chemical cues may be particularly susceptible to rapid divergence between populations. The fact that pronounced premating isolation is observed under conditions that strongly favor chemically based mating decisions suggests that these differences in wing volatiles are likely behaviorally relevant. Definitive demonstration that chemical signals are responsible for reproductive isolation in this species will require manipulative studies that evaluate behavioral responses of both subspecies to experimentally altered volatile profiles.

## Data Availability Statement

Data from this study are available from the Dryad Digital Repository: https://doi.org/10.5061/dryad.269pp30 (McQueen 2018).

## Supplementary Data

Supplementary data are available at *Journal of Insect Science* online.

Supplementary Tables
