# Supplementary material for: Rapid Divergence of Wing Volatile Profiles Between Subspecies of the Butterfly Pieris rapae (Lepidoptera: Pieridae)
Source: J Insect Sci. 2018 Mar 23;18(2):33. doi: 10.1093/jisesa/iey026 (PMC5865527; doi:10.1093/jisesa/iey026)
Supplement: Supplementary Tables [file iey026_suppl_supplementary_tables.docx]

**Supplementary Tables**

| Table S1. Principle component loadings of 13 major components extracted from wings of males and females | | | | | | | | | |
| --- | --- | --- | --- | --- | --- | --- | --- | --- | --- |
|  |  |  |  |  |  |  |  |  |  |
|  |  |  | Not corrected for body size | | |  | Corrected for body size | | |
| No. | Compound |  | *PC1 (49.77% )* | *PC2 (24.60%)* | *PC3 (11.49%)* |  | *PC1 (47.81% )* | *PC2 (19.71%)* | *PC3 (11.43%)* |
| a) All compounds | |  |  |  |  |  |  |  |  |
| 1 | Indole |  | -0.28 | -0.40 | 0.02 |  | -0.30 | -0.38 | 0.06 |
| 2 | Ferrulactone |  | -0.33 | -0.24 | -0.10 |  | -0.33 | -0.24 | -0.04 |
| 3 | Hexahydrofarnecyl acetone | | -0.28 | -0.31 | -0.07 |  | -0.28 | -0.30 | -0.01 |
| 4 | Phytol derivative |  | -0.26 | -0.32 | -0.12 |  | -0.27 | -0.35 | -0.01 |
| 5 | Phytol |  | -0.28 | -0.39 | -0.02 |  | -0.29 | -0.38 | 0.05 |
| 6 | Tricosane |  | 0.30 | -0.18 | -0.46 |  | 0.28 | -0.25 | -0.45 |
| 7 | Pentacosane |  | 0.29 | -0.21 | -0.46 |  | 0.27 | -0.28 | -0.46 |
| 8 | Hexacosane |  | 0.33 | -0.25 | -0.24 |  | 0.31 | -0.30 | -0.21 |
| 9 | Heptacosane |  | 0.33 | -0.27 | 0.08 |  | 0.33 | -0.26 | 0.12 |
| 10 | Octacosane |  | 0.25 | -0.34 | 0.28 |  | 0.26 | -0.30 | 0.33 |
| 11 | Nonacosane |  | 0.22 | -0.30 | 0.45 |  | 0.24 | -0.22 | 0.52 |
| 12 | Hentriacontane |  | 0.29 | -0.10 | 0.27 |  | 0.27 | -0.10 | 0.32 |
| 13 | Cholesterol |  | -0.02 | -0.10 | 0.36 |  | -0.06 | -0.03 | 0.18 |
|  |  |  |  |  |  |  |  |  |  |
| b) Known detectable compounds only | |  | *PC1 (50.44% )* | *PC2 (26.11%)* | *PC3 (15.01%)* |  | *PC1 (53.40% )* | *PC2 (21.88%)* | *PC3 (14.93%)* |
|  |  |  |  |  |  |  |  |  |  |
| 2 | Ferrulactone |  | -0.42 | 0.35 | -0.17 |  | -0.43 | -0.36 | 0.01 |
| 3 | Hexahydrofarnecyl acetone | | -0.37 | 0.46 | -0.06 |  | -0.38 | -0.44 | -0.10 |
| 5 | Phytol |  | -0.34 | 0.49 | 0.10 |  | -0.36 | -0.43 | -0.23 |
| 6 | Tricosane |  | 0.41 | 0.31 | -0.44 |  | 0.38 | -0.44 | 0.33 |
| 7 | Pentacosane |  | 0.40 | 0.34 | -0.44 |  | 0.37 | -0.47 | 0.33 |
| 9 | Heptacosane |  | 0.42 | 0.32 | 0.27 |  | 0.42 | -0.24 | -0.34 |
| 11 | Nonacosane |  | 0.25 | 0.34 | 0.71 |  | 0.28 | -0.10 | -0.78 |

| Table S2. Means and standard deviations of first three principle component scores for each group | | | |
| --- | --- | --- | --- |
| Group | *PC1* | *PC2* | *PC3* |
| 1. All compounds, no body size adjustment | | | |
| *RM* | -3.298 ± 1.008 | -1.355 ± 2.390 | -1.355 ± 2.390 |
| *CM* | -0.572 ± 1.092 | 0.446 ± 1.590 | 0.446 ± 1.590 |
| *RF* | 0.469 ± 0.764 | 1.168 ± 0.737 | 1.168 ± 0.737 |
| *CF* | 2.432 ± 1.838 | -0.346 ± 1.441 | -0.346 ± 1.441 |
| 2. Known detectable compounds, no body size adjustment | | | |
| *RM* | -2.545 ± 0.808 | 1.096 ± 1.659 | -0.461 ± 0.802 |
| *CM* | -0.471 ± 0.725 | -0.429 ± 1.029 | 0.297 ± 1.176 |
| *RF* | 0.414 ± 0.548 | -0.917 ± 0.518 | 0.189 ± 0.587 |
| *CF* | 1.853 ± 1.551 | 0.305 ± 1.268 | -0.043 ± 1.318 |
| 3. All compounds, with adjustment for body size | | | |
| *RM* | -3.599 ± 0.882 | -1.309 ± 1.917 | -0.301 ± 0.890 |
| *CM* | -0.839 ± 0.945 | 0.919 ± 1.159 | 0.306 ± 1.179 |
| *RF* | 1.007 ± 0.692 | 0.800 ± 0.641 | 0.402 ± 0.704 |
| *CF* | 2.381 ± 1.686 | -0.408 ± 1.56 | -0.352 ± 1.702 |
| 4. Known detectable compounds, with adjustment for body size | | | |
| *RM* | -2.770 ± 0.720 | -1.082 ± 1.148 | 0.083 ±1.036 |
| *CM* | -0.603 ± 0.681 | 0.667 ± 0.571 | 0.089 ± 1.207 |
| *RF* | 0.750 ± 0.519 | 0.758 ± 0.378 | -0.274 ± 0.667 |
| *CF* | 1.823 ± 1.422 | -0.353 ± 1.496 | 0.111 ± 1.204 |
| RF = *P. r. rapae* females, RM = *P. r. rapae* males, CF = *P. r. crucivora* females, CM = *P. r. crucivora* males. | | | |

| Table S3. Group prediction using linear discriminants | | | | | |
| --- | --- | --- | --- | --- | --- |
|  | Subspecies and sex | % Correct Prediction | Cross-validation | % Correct Prediction | Cross-validation |
|  |  |  |  |  |  |
|  |  | Not adjusted for body size | | With body size adjustment | |
| All identified compounds | *P. r. crucivora*, female | 73% | 60% | 73% | 60% |
|  | *P. r. rapae*, female | 92% | 92% | 92% | 85% |
|  | *P. r. crucivora*, male | 100% | 91% | 100% | 91% |
|  | *P. r. rapae*, male | 91% | 73% | 91% | 73% |
| Known detectable Compounds | *P. r. crucivora*, female | 73% | 67% | 87% | 47% |
|  | *P. r. rapae*, female | 92% | 69% | 92% | 77% |
|  | *P. r. crucivora*, male | 82% | 73% | 91% | 91% |
|  | *P. r. rapae*, male | 91% | 82% | 91% | 91% |

| Table S4. Mahalanobis distances and MANOVAs of first three linear discriminants | | | | | |
| --- | --- | --- | --- | --- | --- |
| *Comparison* | *MD* | *Df* | *Wilks* λ | *approx F* | *p-value** |
| 1. All identified compounds, not adjusted for body size | | | | |  |
| *RM x CM* | 60.1 | 1 | 0.193 | 25.0 | 7.25E-06 |
| *RM x RF* | 215 | 1 | 0.0888 | 68.4 | 6.52E-10 |
| *CM x RF* | 79.4 | 1 | 0.117 | 50.1 | 1.05E-08 |
| *CM x CF* | 94.7 | 1 | 0.0995 | 66.4 | 2.09E-10 |
| *RM x CF* | 232 | 1 | 0.0788 | 85.8 | 1.62E-11 |
| *RF x CF* | 5.29 | 1 | 0.548 | 6.60 | 0.0124 |
| 2. Known detectable compounds only, not adjusted for body size | | | | |  |
| *RM x CM* | 58.8 | 1 | 0.251 | 17.9 | 7.48E-05 |
| *RM x RF* | 105 | 1 | 0.112 | 53.0 | 6.41E-09 |
| *CM x RF* | 8.53 | 1 | 0.213 | 24.7 | 3.74E-06 |
| *CM x CF* | 9.28 | 1 | 0.219 | 26.2 | 1.14E-06 |
| *RM x CF* | 98.2 | 1 | 0.101 | 65.6 | 2.36E-10 |
| *RF x CF* | 3.23 | 1 | 0.665 | 4.04 | 0.111 |
| 3. All identified compounds, with overall body size adjustment | | | | |  |
| *RM x CM* | 74.0 | 1 | 0.170 | 29.3 | 2.32E-06 |
| *RM x RF* | 263 | 1 | 0.0782 | 78.6 | 1.83E-10 |
| *CM x RF* | 91.8 | 1 | 0.0830 | 73.6 | 3.32E-10 |
| *CM x CF* | 84.9 | 1 | 0.0937 | 70.9 | 1.08E-10 |
| *RM x CF* | 243 | 1 | 0.0756 | 89.6 | 1.04E-11 |
| *RF x CF* | 5.03 | 1 | 0.578 | 5.83 | 0.0231 |
| 4. Known detectable compounds only, with overall body size adjustment | | | | | |
| *RM x CM* | 75.6 | 1 | 0.225 | 20.7 | 2.74E-05 |
| *RM x RF* | 151 | 1 | 0.0786 | 78.2 | 1.92E-10 |
| *CM x RF* | 14.9 | 1 | 0.270 | 18.0 | 4.00E-05 |
| *CM x CF* | 12.1 | 1 | 0.252 | 21.7 | 5.40E-06 |
| *RM x CF* | 132 | 1 | 0.0799 | 84.5 | 1.89E-11 |
| *RF x CF* | 3.52 | 1 | 0.755 | 2.60 | 0.452 |
| *Bonferroni adjusted  RF = *P. r. rapae* females, RM = *P. r. rapae* males, CF = *P. r. crucivora* females, CM = *P. r. crucivora* males. | | | | | |
